# Validation of a Homogeneous Incremental Centrifugal Liquid Sedimentation Method for Size Analysis of Silica (Nano)particles

**DOI:** 10.3390/ma13173806

**Published:** 2020-08-28

**Authors:** Jesús Manuel Antúnez Domínguez, Yannic Ramaye, Marta Dabrio, Vikram Kestens

**Affiliations:** European Commission, Joint Research Centre (JRC), 2440 Geel, Belgium; jesusantunezdominguez@hotmail.com (J.M.A.D.); yannic.ramaye@ec.europa.eu (Y.R.); marta.dabrio@ec.europa.eu (M.D.)

**Keywords:** centrifugal liquid sedimentation, measurement uncertainty, method validation, nanoparticles, particle size analysis, reference materials, silica

## Abstract

Silica nanoparticles display many unique physicochemical properties that make them desirable for use in a wide variety of consumer products and composite materials. Accurately measuring the size of these nanoparticles is important for achieving the desired nanoscale functionality of the final product and for regulatory compliances. This study covers the validation of a centrifugal liquid sedimentation method for accurate measurement of the Stokes diameter of silica particles with a near-spherical shape and dimensions in the nanometer and sub-nanometer scale range. The validated method provided unbiased results in the range of 50 nm to 200 nm, with a lower limit of detection of ≤20 nm. The relative standard uncertainties for precision, quantified in terms of repeatability and day-to-day variation, ranged from 0.2% to 1.0% and from <0.1% to 0.5%, respectively. The standard uncertainty for trueness was assessed at 4.6%. Within its working range, the method was found robust with respect to the type of cuvette, light factor, operator, and for defining the meniscus of the sample suspension. Finally, a relative expanded measurement uncertainty of 10% confirmed the satisfactory performance of the method.

## 1. Introduction

Nanoparticles and nanomaterials in general are at the leading edge of the rapidly developing field of nanotechnology. The small particle size confers them unique physical and chemical properties compared to their bulk counterparts. As a result, nanoparticles have become ubiquitous in our daily lives [1,2,3].

Along with the increased use of nanoparticles, concerns emerge about occupational exposure and potential adverse health effects [4,5]. In safeguarding public health and the environment in the European Union (EU), horizontal and vertical legislation has been put in place [6,7,8]. This legislation is based on the European Commission’s Recommendation (2011/696/EU) on the definition of nanomaterial [9]. Although the definition is overarching in nature, a practical implementation requires validated measurement procedures for particle size analysis, which should include the use of fit-for-purpose certified reference materials.

Over the last decade, significant efforts have been made to develop and validate methods for accurate sizing of monodisperse and near-spherical nanoparticles. These methods include popular techniques such as electron microscopy [10,11,12], dynamic light scattering [13,14,15], disc-type centrifugal liquid sedimentation [13,14,15,16,17], and particle tracking analysis [18,19,20,21]. Method validation is important to demonstrate that a method is working as expected and therefore it is a key step during the process of standardization. Thanks to previous intra- and interlaboratory validation studies, different particle size analysis methods have been successfully standardized by, for instance, the Technical Committees (TC) on Particle Characterization (TC 24) and Nanotechnologies (TC 229) of the International Organization for Standardization (ISO) [22,23,24,25]. Standardized methods are important as they ensure reliability of results and provide benchmarks for comparing measurement results across space and time.

A technique that has gained wide acceptance for measuring the size of nano- and microparticles is centrifugal liquid sedimentation (CLS) or analytical centrifugation [25]. The technique owes its popularity to its high resolution and precision with which particles that differ in size and/or density can be separated from one another. The CLS technique has been commercialized in two distinctly different instrumental designs [26]. The variant implemented most commonly makes use of an optically clear and hollow disc partly filled with a sucrose-based fluid that exhibits a radial density (and viscosity) gradient. A small volume (~few microliters) of a sample suspension is injected at the center of the disc. Because all particles start their sedimentation from the same radial position, the disc-type CLS technique is referred to as the line-start incremental method. The combination of a high angular speed (up to 24,000 rev/min or 27,400 g) and a density gradient allows accurate measurement of the size of silica nanoparticles with diameters as small as 20 nm [13]. The second instrument setup employs a classical rotor system in which individual sample cells are placed and rotated at a relatively low speed (up to 4000 rev/min or 2300 g). As the particles are initially uniformly distributed in the sample suspension, the cuvette-type CLS technique is also known as the homogeneous incremental method. Despite the two different instrument setups, both systems apply the measurement principle of Stokes’ law

(1) $$x_{\mathrm{St}}=\;\sqrt{\frac{18\eta \ln\left(M/S\right)}{\left(\rho_{p}-\rho_{l}\right)\omega^{2}t_{p}}}$$

where *x*_St_ is the Stokes diameter of the particle settled and detected after time *t*_p_, *η* is the dynamic viscosity of the liquid, *M* and *S* are respectively the measurement position and the initial position of the meniscus in the liquid (in case of cuvette-type CLS) or the radius of rotation at the inner liquid surface of the density gradient (in case of disc-type CLS), *ρ*_p_ and *ρ*_l_ are the respective densities of the particles and liquid, ω is the rotational or angular speed of the rotor or the disc.

Besides the difference in design and measurement setup, the cuvette-type CLS instrument differs essentially from the disc-type instrument in that its sedimentation time scale does not require calibration with reference particles of known size and effective density. Calibrating the sedimentation time scale of disc-type CLS instruments is common practice as it is the most user-friendly approach to cope efficiently and quantitatively with the combined effect of several input quantities (i.e., *η*, *ρ*_l_, *S*) which continuously change throughout a measurement sequence, due to the injection of test samples and calibrant samples. However, calibration poses risks when calibrants are not well characterized [27,28]. Other advantages that makes the cuvette-type CLS method (further referred to as cuvette-CLS) standing out compared to the disc-CLS method is the absence of a density gradient which makes it possible to analyze the particles in their original state and dispersant.

Another technique that is part of the CLS family, and which should be mentioned for the sake of completeness, is analytical ultracentrifugation (AUC). This technique has similarities with the previously described cuvette-CLS in that it also uses sample cuvettes and operates in the homogeneous incremental mode. The main strength of AUC is that it can operate at a much higher relative centrifugal field (up to 60,000 g) which makes the technique even suitable to analyze the size, shape, and interactions of macromolecules [29] and to distinguish between particle monomers, dimers, and small agglomerates [30]. Particle size analysis by means of AUC is, however, much less implemented in routine laboratories as this type of centrifuge comes at a much higher cost than cuvette-CLS instruments.

In the field of particle size analysis, measurements are traditionally performed using the popular dynamic light scattering (DLS) technique or electron microscopy. While DLS is a user-friendly technique, it is a low-resolution method that often cannot separate particles of significantly different sizes [31]. On the other hand, electron microscopy allows single particle observation allowing to discriminate between different particle populations. However, electron microscopes are expensive instruments which are often labor intensive in operation and require highly skilled operators. Also, the success of a reliable quantitative assessment, especially for polydisperse samples, depends on well-prepared specimens and validated image analysis strategies [32].

This paper presents the results of an in-house conducted validation study of a user-friendly, cost-efficient, and high-resolution cuvette-CLS method developed for particle size analysis of monodisperse and bimodal samples of near-spherical silica particles with diameters in the range of 20 nm to 200 nm. The design and conduct of the validation study followed guidelines recommended by EURACHEM [33] while measurement uncertainties were estimated according to ISO/IEC Guide 98-3 [34]. The uncertainty budget allows to compare the performance of the validated cuvette-CLS method with other previously validated methods [12,13,14,19,31]. The validated method will be used by the European Commission’s Joint Research Centre (EC-JRC) during the production of future colloidal silica CRMs. In addition, the present study can be considered as a practical guide for others who are in need of validating measurement methods.

## 2. Materials and Methods

### 2.1. Reference Materials

The water-based colloidal silica CRMs and (non-certified) RMs used in this study are presented in Table 1. Materials coded as ‘ERM’ and ‘NS’ were supplied by the JRC (Geel, Belgium) and the htt Group (Munich, Germany), respectively. With the exception of ERM-FD102, which has a particle size distribution (PSD) consisting of two distinct peaks, all materials are monodisperse. The assigned modal particle diameter values include related expanded uncertainties corresponding to a confidence level of about 95%. For the ‘NS’-type materials, the particle size values are area-equivalent diameters determined by transmission electron microscopy. The size values assigned to the ‘ERM’-type materials are Stokes diameters determined by disc- and cuvette-CLS. Both RMs and CRMs are stable and homogeneous with respect to their assigned property values. Furthermore, CRMs represent a higher metrological reference standard as their assigned property/certified values are characterized using metrologically valid procedures which make them reliable estimates of the true values [35]. As a result, CRMs can be used for assessing the trueness (i.e., performance parameter for bias or systematic error) of a method, while RMs may be used only for relative assessments such as precision testing. All CRMs were analyzed as-received while the RMs were diluted using purified water (18.2 MΩ cm at 25 °C) prior to analysis.

Bimodal samples were prepared by mixing two monodisperse materials thereby covering the particle size range of 50 nm to 200 nm in diameter (Table 2). Three milliliters of each monodisperse suspension (with or without dilution, as required per material) were mixed together.

### 2.2. Instrumentation

Cuvette-CLS measurements were performed with a LUMiSizer 650 cuvette-type analytical photocentrifuge (LUM GmbH, Berlin, Germany). The instrument is equipped with a twelve-place rotor, mounted on a vertical shaft and driven by a variable speed motor that can be operated at 200 rev/min up to 4000 rev/min. The sample cuvettes are placed horizontally into the channels of the rotor. The optical system consists of a blue LED source (470 nm wavelength) and a turbidity CCD-line sensor with 2048 elements that detects the intensity of the light transmitted by the sample suspension as a function of time and position across the entire length of the cuvette. The detector has an extinction working range of 0.1–4.0 (expressed as light factor). Furthermore, the instrument is equipped with a Pt 100 temperature sensor and a controller that allows setting and maintaining a stable temperature (±1 °C) in the range of 4 °C to 40 °C. The samples were analyzed in polycarbonate rectangular cuvettes with 2 mm or 10 mm optical path lengths. The former were filled with 0.4 mL of suspension, the latter with 1.5 mL. The settings applied for data acquisition are given in the section discussing the method development part. Data processing and analysis was performed in the ‘constant position’ mode using the SEPView^TM^ 6.0 instrument software. The constant position mode used a set of three measurement positions of 1.0 mm width applied along the length of the cuvette part containing the sample suspension [36]. Particle size distributions were calculated for each measurement zone and averaged as to obtain a robust light extinction-weighted PSD representative for the entire sample. The representation and calculation of the PSDs’ characteristic parameters was in accordance to ISO 9276-1 and ISO 9276-2 [37,38]. The characteristic parameters were estimated from lognormal functions fitted to linearly spaced PSDs. Detailed information about data acquisition and data processing procedures used by the SEPView^TM^ 6.0 software are explained elsewhere [39].

### 2.3. Method Development and Optimization

A preliminary investigation was conducted to establish a suitable method for size analysis of near-spherical silica particles with external dimensions in the range of 20 nm to 200 nm and mass fractions in the range of 0.1 g/kg to 10 g/kg. Because of the relatively broad size range, this study mainly focused on optimizing those method parameters that can influence the efficiency of particle detection and analysis. The parameters examined were: type of cuvette, particle mass fraction and dilution, data acquisition time, light factor (LF), defining the measurement positions and sample meniscus and subtraction of the background signal.

Experiments were conducted such that only a single parameter was varied at a time while keeping the other variables constant. One-way analysis of variance (ANOVA) was used to evaluate the differences between group means.

An extensive discussion of the results obtained during the method development study is available as Supplementary Materials. An overview of the parameters, and their optimized levels, is shown in Table 3.

### 2.4. Method Validation

The method performance parameters evaluated were robustness, limit of detection, limit of quantification, working range, selectivity, repeatability, intermediate precision, or day-to-day variation, and trueness. Other common parameters such as calibration and linearity, and stability of samples, were considered not relevant due to the nature and measurement principle of the cuvette-CLS method. The design of the validation study followed guidelines recommended by EURACHEM [33]. The performance of the validated method was assessed against predefined criteria. These criteria were established based on results from validation studies conducted previously on other particle size analysis methods such as dynamic light scattering, disc-type CLS, PTA, and TEM [12,13,14,19]. The method is considered fit-for-purpose if the predefined criteria are met. The validation parameters, the type of experiments, and the predefined performance criteria are summarized in Table A1 of Appendix A.

With the exception of NS-0020A and ERM-FD306, the RMs and CRMs listed in Table 1 were systematically analyzed applying the cuvette-CLS optimized method (Table 3). For each material, a total of 20 measurements (independent replicates) were conducted under repeatability and intermediate precision conditions according to a nested experimental design that equally distributed the 20 measurements over five days. One-way ANOVA was used to separate the variances within and between groups. The relative standard deviations for repeatability (intra-day) and for intermediate precision (inter-day) were calculated according to

(2) $$RSD_{r}=\;100\cdot \frac{\sqrt{MSW}}{y_{m}}$$

(3) $$RSD_{\mathrm{ip}}=\;100\cdot \frac{\sqrt{\frac{MSB-MSW}{n_{r}}}}{y_{m}}$$

where, *RSD*_r_ and *RSD*_ip_ are the relative standard deviations for repeatability and intermediate precision, *MSW* and *MSB* are the mean squares within and between groups (i.e., days), *n*_r_ is the number of measurement replicates (four) per day, and *y*_m_ is the arithmetic average calculated from the 20 replicate results.

If *MSB* < *MSW*, then Equation (3) loses its validity due to the negative number under the square root. In such a situation, an alternative approach was applied to calculate the relative variability between measurement days, $RSD_{\mathrm{ip}}^{*}$ [40]

(4) $$RSD_{\mathrm{ip}}^{*}=\;100\cdot \frac{\sqrt{\frac{MSB-MSW}{n_{r}}+\frac{MSWe^{-\left(\frac{MSB}{MSW}\right)}}{n_{r}}}}{y_{m}}$$

Measurement uncertainties were estimated using a combination of bottom-up and top-down approaches [33], including standard uncertainties (at a confidence level of about 68%) for the precision and trueness components of the validated method.

The *RSDs*, which provide a quantitative expression of the repeatability and intermediate precision of the method, were considered as reliable estimates for the relative standard uncertainties for repeatability (*u*_r_) and intermediate precision (*u*_ip_). On that basis, the relative standard uncertainty for precision (*u*_prec_) was estimated following Equation (5), in which *RSD*_ip_ may be replaced by $RSD_{\mathrm{ip}}^{*}$, if applicable. The standard uncertainty for precision is estimated from results of four sample replicates analyzed under repeatability conditions (i.e., on a single measurement day, *n*_d_ = 1)

(5) $$u_{\mathrm{prec}}=\;\sqrt{\frac{RSD_{r}^{2}}{n_{r}}+\frac{RSD_{\mathrm{ip}}^{2}}{n_{d}}}$$

The trueness of the cuvette-CLS method was quantitatively assessed in terms of experimental bias (Δ_bias_), which is the absolute difference between the certified value of a CRM, or an accepted reference value, and the average calculated from the modal particle size values of the replicate measurement results.

Applying the procedure recommended by accredited CRM producers [41], the experimental bias is considered significant on a confidence level of 95% if Δ_bias_ > 2 × *u*_t_

(6) $$u_{t}=\sqrt{u_{\mathrm{meas}}^{2}+u_{\mathrm{CRM}}^{2}}$$

where *u*_t_ is the relative standard uncertainty for trueness, *u*_meas_ is the relative standard uncertainty associated to the mean of the modal particle size experimental results obtained for the CRM, and *u*_CRM_ is the relative standard uncertainty of the certified value. The latter is normally available from the CRM certificate.

In principle, the determination of trueness is established by analyzing CRMs. However, for diameters of >100 nm, suitable colloidal silica CRMs are not available. To evaluate the performance of the cuvette-CLS method in the upper particle size range, results obtained on the (non-certified) ‘NS’-type RMs were compared with their assigned reference values. Agreement within 9% (relative difference, *RD*) was considered acceptable.

The relative standard uncertainties of the measurement results obtained on the CRMs correspond to the expression of measurement precision and are estimated as indicated in Equation (5), with that difference that *n*_d_ = 5 as these results were obtained over five days.

The expanded measurement uncertainties (*U*) for Stokes diameters (mode, median, harmonic mean) were obtained by combining the relative standard uncertainties of the precision and trueness using the usual root-mean-square manner, and following the procedures described in ISO/IEC Guide 98-3 [34]. The estimated expanded uncertainties are valid for the average of four replicate results all obtained under repeatability conditions on one day. A coverage factor, *k* = 2, was used to express the uncertainties on an approximate 95% confidence interval.

(7) $$U\;=\;k\cdot \sqrt{u_{\mathrm{prec}}^{2}+u_{t}^{2}}$$

Whether particles can be effectively detected and measured depends on a combination of instrumental parameters and material/sample properties. By fixing the key instrumental parameters such as the angular velocity (i.e., 4000 rev/min) and the wavelength of the incident light beam (i.e., 470 nm), the limit of detection (LOD) and limit of quantification (LOQ) could be derived from the experimental results and from pre-defined limits related to statistical significance and *RSD*_r_ (Appendix A).

To investigate the cuvette-CLS’s selectivity in terms of the capability to distinguish between particles that differ in size (within the given working range), different bimodal mixtures of monodisperse silica CRMs/RMs were prepared (Table 2) and analyzed in quadruplicate under repeatability conditions. Also, the bimodal silica CRM, ERM-FD102, was analyzed in this study.

To allow a quantitative assessment, the resolution (*R*_s_) was expressed using Equation (8) [42]

(8) $$R_{s}=\;1.18\cdot \frac{X_{c2}-X_{c1}}{W_{0.5,1}+W_{0.5,2}}$$

where, *X*_c1_ and *X*_c2_ correspond to the local maxima of peak 1 and peak 2 present in the PSD, *W*_0.5,1_ and *W*_0.5,2_ are the peak widths measured at half-height, the value of 1.18 is a constant factor which adjusts for the difference in width and the half-height of Gaussian peaks.

The *R*_s_ value is a relative measure of how well two neighboring peaks are separated. An *R*_s_ value of ≥1.5 indicates a baseline resolved separation. As the instrument software did not allow calculating the width at half-height values, the PSD data were exported and manually fitted with amplitude-based Gaussian functions using the software Origin 2019b (OriginLab Corp., Northampton, MA, USA). It must be noted that particle size distributions are usually best described by lognormal fits, as they represent well the tails. However, for narrow and symmetric peaks, Gaussian fits can provide equally accurate peak characteristic data.

Robustness refers to the method’s suitability and capacity to obtain similar results when perturbed by deliberate and small variations in procedural parameters (temperature, setting of the sample meniscus, light factor). The influence of these parameters was examined during the development stage of the method by analyzing ERM-FD305 under repeatability conditions. During the method validation process, the robustness of the optimized method was further checked against different operators. For each of the selected silica materials, two out of the five measurement days were conducted by the second analyst.

## 3. Results

### 3.1. Precision

The repeatability and intermediate precision were assessed using the results of the monodisperse silica CRMs and RMs analyzed according to a nested experimental design (four replicates per day and five measurement days). Table 4 gives an overview of the mean results of the modal, median, and harmonic mean diameters, the relative standard deviations for repeatability and intermediate precision, and the associated combined relative standard uncertainties for precision.

### 3.2. Trueness, LOD, LOQ, Working Range

The trueness of the method was assessed by evaluating the significance of the bias calculated from the certified modal Stokes diameter values and the mean values of the modal particle diameter experimental results. The results of the trueness evaluation and of the comparative analysis of non-certified RMs are summarized in Table 5. These results were also employed to establish the LOD, LOQ, and working range (in terms of particle size).

### 3.3. Robustness

The robustness of the method was mainly assessed during the method development phase by examining the central tendency measures (mode, harmonic mean, median) of the light extinction-weighted PSDs obtained for selected silica (C)RMs, while varying slightly selected method parameters. The results of the robustness study are shown along with the examined parameters and their levels in Table 6. A more detailed overview is given in the Supplementary Materials.

### 3.4. Selectivity

**Figure 1:**
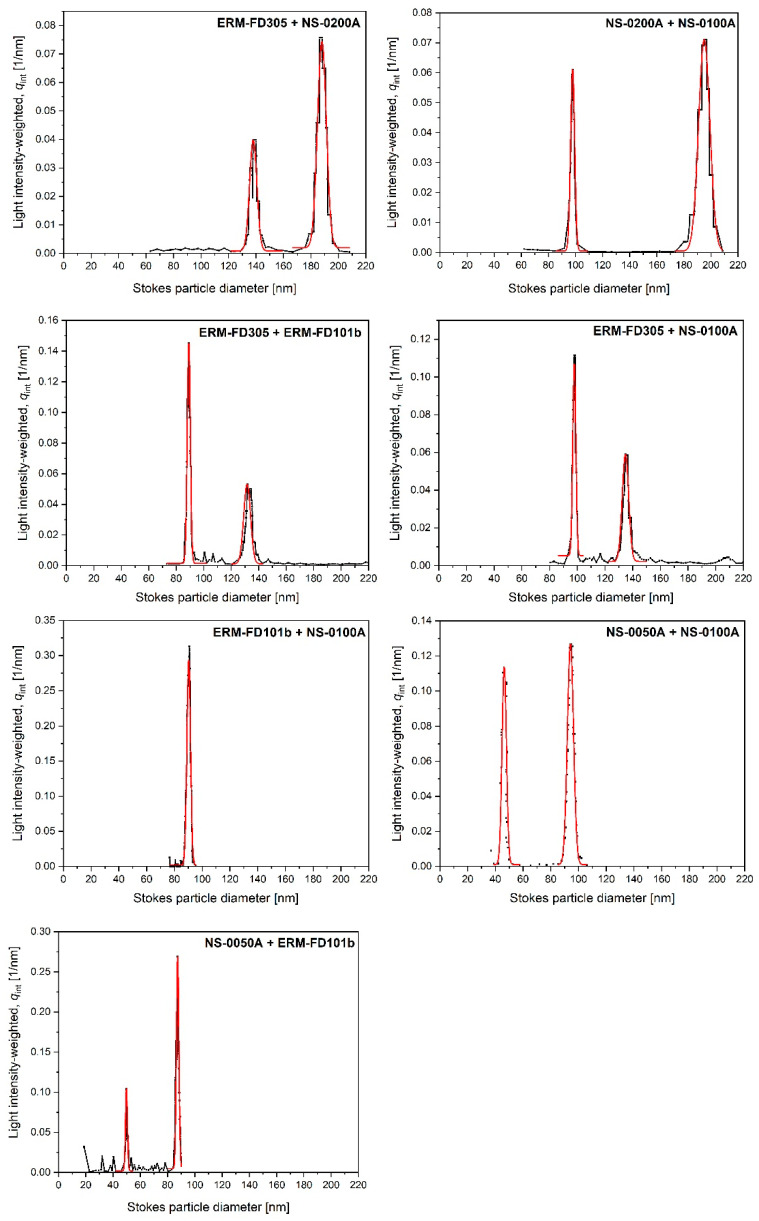
Representative examples of light intensity-weighted PSDs of bimodal colloidal silica mixtures obtained by cuvette-CLS, red curves are Gaussian fits.

The capability of the cuvette-CLS method to distinguish quantitatively between two populations of silica particles that differ in size, was investigated by analyzing different bimodal mixtures of monodisperse silica CRMs/RMs (Table 2) and the bimodal silica CRM (ERM-FD102). An overview of the particle size results of the peaks’ local maxima, their ratio and *R*_S_ values, is given in Table 7. Representative examples of the PSDs with overlaid Gaussian fits are depicted in Figure 1.

### 3.5. Measurement Uncertainty

**Figure 2:**
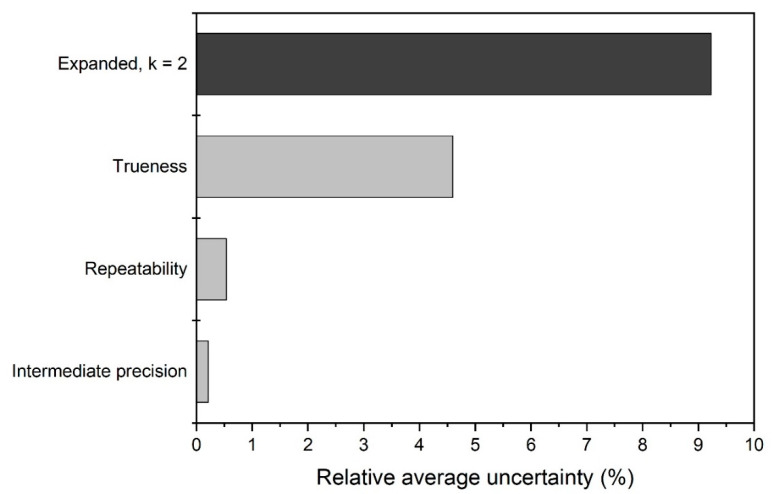
Relative average standard and expanded measurement uncertainties (%) estimated for particle size results from the validated cuvette-CLS method.

A graphical representation of the uncertainty budget of the validated method is depicted in Figure 2. The uncertainties are averages which account for the three types of central tendencies in the range of 50 nm to 200 nm.

## 4. Discussion

### 4.1. Method Development and Optimization

Prior to the validation study, a suitable cuvette-CLS method for measuring the size of silica particles with diameters in the range of 20 nm to 200 nm was developed by conducting a series of experiments with varying method conditions. A brief summary of the method development study is given in the following paragraphs. A more detailed discussion is available as Supplementary Materials.

Tests with two types of measurement cuvettes (i.e., 2 mm and 10 mm optical path length) gave similar particle size results for ERM-FD305 (Table S1). The use of cuvettes with different optical path lengths increases the versatility of the method to analyze samples of different optical turbidity.

For highly concentrated samples, i.e., when the level of the first data profile is significantly below 30% of transmission (Figure S1), dilution with a suitable diluent would be required to bring the initial transmission level within the recommended range of 30% to 60%. The results shown in Table S2 demonstrate that the analyzed colloidal silica RMs can be safely diluted without compromising the integrity of the material. While it is good practice to always verify the possible effect of sample dilution, the ability to dilute samples can also be beneficial when the available amount of the original sample is too small for the available sample cuvettes.

One of the most crucial parameters in analytical centrifugation is the measurement or data acquisition time. Acquisition times set too short will result in PSDs that are incomplete and biased. On the other hand, acquisition times set unnecessarily long reduce sample throughput. Using selected monodisperse silica RMs the different segments (i.e., number of cycles and time intervals) of the data acquisition program were optimized to ensure that silica particles with diameters in the range of 20 nm to 200 nm can be detected (Tables S3 and S4). The established program corresponds to a total acquisition time of 15 h.

A general limitation of measurement equipment that apply the principle of light extinction is saturation of the photodetector. For the cuvette-CLS method, this may occur when samples have a highly translucent appearance and thus only a small fraction of the incident light is extinct by the sample suspension. To avoid saturation of the detector when analyzing such type of samples, the intensity of the incident light beam can be reduced by applying a lower value for the light factor. According to the instrument manufacturer, a default value of 0.7 is suitable for most types of colloidal systems. Since silica nanoparticles are known to be weak scatterers of light, the effect of light factor values of 0.7 and 0.25 was studied (Table S5). For the tested silica RMs, it was found that the reduced light factor value has no significant impact on the main central tendency values of the PSDs. For ERM-FD305, which is slightly turbid, the possible impact of a light factor value of 1.0 was additionally tested. Also for this material, no significant effect on the calculated particle size could be demonstrated.

When the centrifugation program has completed, the acquired data is converted to light extinction-weighted PSDs. To do so, the sedimentation data recorded in the light transmission profiles is analyzed at three default measurement zones distributed over the lower part of the sample cuvette, i.e., at a distance of (123.0 ± 0.5) mm, (125.0 ± 0.5) mm and (127.0 ± 0.5) mm from the center of revolution. To investigate the effect of the measurement position, PSDs were calculated by selecting measurement zones in the range of 110 mm to 127 mm. The results in Table S7 show a systematic increase of the calculated particle size with the distance from the center of rotation. The difference in particle size calculated for the two extreme positions is about 3%. To flatten the effect of the positive trend, it was decided to use the average of the three default measurement zones (as recommended by the instrument manufacturer).

The start of the sedimentation process is given by the sample meniscus (Figure S1). Defining the meniscus can be subject to variation as the local minima of the different transmission profiles do not always exactly overlap. To evaluate the effect of the meniscus setting, PSDs were calculated using the first, middle, and last transmission profiles, respectively. It was found that the profile used for the meniscus setting does not significantly affect the central tendency values of the PSDs (Table S6). Nevertheless, to eliminate the precision component of the meniscus setting process it was decided that the meniscus will be defined from the last acquired transmission profile.

Finally, the method development study also showed that the time-independent background signal can be subtracted by using either the last acquired transmission profile or using the profiles obtained for a blank sample (Table S8).

### 4.2. Precision

As the validated method is intended for single-laboratory use, the precision profile of the method was characterized in terms of repeatability (within-day variation) and intermediate precision (day-to-day variation). The between-laboratory precision, or reproducibility, was beyond the scope of the present study.

From the results listed in Table 4, it can be concluded that silica nanoparticles smaller than 50 nm in nominal diameter compromise the repeatability of the method, as for those particles the *RSD*_r_ values greatly exceeded the 1.5% repeatability limit. For diameters in the range of 50 nm to 200 nm, the method performed satisfactorily. The between-day variation was below 1% and showed to be independent from the measured particle size. For the particle size range of 50 nm to 200 nm, the overall relative standard uncertainty for precision, *u*_prec_, varied between 0.1% and 0.7%. Analyzing more than four replicates is advantageous as it reduces the repeatability uncertainty. However, since repeatability and intermediate precision contribute equally to *u*_prec_, the latter will not improve significantly (cf. Equation (5)).

### 4.3. Trueness

The trueness of the method was assessed by analyzing the certified reference materials ERM-FD100, ERM-FD304, and ERM-FD101b. As seen from the results in Table 5, significant biases were obtained for ERM-FD100 and ERM-FD304 indicating that the method is unable to accurately measure the Stokes diameter of 30 nm silica nanoparticles, and smaller. These results complement the previous results of the precision evaluation where significantly higher *RSD*_r_ values were obtained for the same CRMs. On the other hand, the bias for ERM-FD101b was found not significant. To fully understand and assess the trueness of the method across its entire working range, additional experiments on colloidal silica CRMs with particle sizes of 100 nm and more would be needed. As such CRMs are currently not available, the results obtained on the non-certified RMs, NS-0050A, NS-0100A, NS-0150A, and NS-0200A, were used to determine the measurement performance of the method in the upper particle size range. To judge whether these measurement results were sufficiently close to the assigned reference values of the RMs, a relative difference (*RD*) of maximum 9% was considered as acceptable. This acceptance criterion was established based on the 9.2% relative expanded uncertainty of the certified value of ERM-FD101b. Since the RMs embody particle size values that were assigned by transmission electron microscopy, no metrologically sound conclusions can be made on trueness from the obtained Stokes diameters measured by cuvette-CLS. Nevertheless, as these silica particles are highly monodisperse and do not swell in a liquid environment, a meaningful comparison can be justified, even for different measurands.

As demonstrated by the results in Table 5, the *RD* values were within the acceptance limit, showing that the cuvette-CLS method yields accurate results in the particle size range of 50 nm to 200 nm and in the particle mass fraction range of 0.1 g/kg to 2.5 g/kg.

### 4.4. Limit of Detection and Limit of Quantification

For chemical methods, the lower limits of detection and quantification are the smallest analyte concentrations that can be detected and measured, respectively. They are often quantified as 3-times and 10-times the standard deviation calculated from results obtained by measuring blank samples [33]. For physical methods that measure the size of particles in suspension, such as the described cuvette-CLS method, measuring blank samples is not relevant as the background signal of a particle-free sample cannot be meaningfully related to the method’s particle size measurement performance. In addition, the limits of detection and quantification do not solely depend on the target measurand (i.e., particle size measured as equivalent diameter), but they also depend on the mass fraction of the particles. For instance, at a given mass fraction, the detection/measurement of small nanoparticles can be more challenging than for larger particles of a similar composition. However, at sufficiently high mass fractions the small nanoparticles may become easier to detect/measure while the larger particles can become undetectable/unmeasurable at very low mass fractions. For the cuvette-CLS method, which has been developed and validated for a relatively broad particle size range, it is thus not straightforward to experimentally determine separate limits of detection and quantification for particle mass fraction. Instead, based on the dilution experiments conducted during the method development phase (Table S2) and the particle mass fractions of the colloidal silica RM test samples successfully analyzed during the validation study (Table 1), a particle mass fraction of 0.1 g/kg to 2.5 g/kg can be regarded as a ‘safe range’ (for silica). When transferring the validated method to ‘unknown’ colloidal silica materials, one should nevertheless carefully verify whether the mass fraction of the given sample (with nominal diameters in the range of 50 nm to 200 nm) is suitable. A simple and fast way to do so is by ensuring that the transmission signal of the first acquired data profile is in the range of 30% to 60% (Figure S2) and that the relative standard deviation of the particle size results is ≤1.5% (acceptance criterion for repeatability).

For the cuvette-CLS method, the particle size results from the repeatability and trueness studies were used to estimate the lower limit of detection (LLOD_d_), the lower limit of quantification (LLOQ_d_), and the upper limit of quantification (ULOQ_d_). The subscript ‘d’ denotes that the limit values are defined as Stokes diameters.

As can be seen from the *RSD*_r_ results (Table 4), the repeatability of the method is satisfactory in the range of 50 nm to 200 nm, as the *RSD*_r_ values for those particles are ≤1.5%. For the same particle size range, it was found that the experimental result agreed with either the certified value or the assigned non-certified values within a relative difference of 9%. Based on both types of evaluations, the LLOQ_d_ and ULOQ_d_ are set at nominally 50 nm and 200 nm, respectively. For silica nanoparticles smaller than 50 nm in diameter, the repeatability significantly deteriorates. However, their *RSD*_r_ values are still ≤5.0%, and therefore they can still be detected reliably. As silica nanoparticles with diameters smaller than 20 nm were not available to further test the limits experimentally, the LLOD_d_ is set conservatively at nominally 20 nm.

### 4.5. Linearity and Calibration

The measurement signal response of the cuvette-CLS method does not require calibration with particle size CRMs. As a result, linearity, as known in the field of traditional chemical analysis, is not applicable. There are, of course, a number of instrument parameters (e.g., temperature sensor, rotor speed, alignment of the optical system) which need to be verified with properly calibrated tools, but these parameters, which are checked on a regular basis with SI-traceably calibrated devices, are assumed not to contribute significantly to measurement uncertainty.

### 4.6. Robustness

The robustness of the optimized method was examined by evaluating the influence of small variations in selected method parameters. These parameters were related to the temperature, the LF and the position of the sample meniscus. In general, the *F*-statistic in ANOVA did not flag significant differences between group means indicating that the method is robust against the influence of minor modifications in operational conditions. Despite that the analytical centrifuge is equipped with a temperature controller and that results are automatically corrected for deviation from set temperature, a systematic trend of decreasing calculated Stokes diameters was, nevertheless, noted for increasing temperature. At 26 °C the trend resulted in harmonic mean and median particle diameter results which were significantly lower than results obtained at 24 °C (−1.0%) and 25 °C (−0.7%). During a previous study, it was concluded that a variation of about 1 °C affects the measured Stokes diameter by approximately 2.5% [28]. This uncertainty, which in the present study is assumed to be fully represented by the precision uncertainty, largely covers the experimental deviations. Therefore, the two results that were identified as statistical outliers are technically acceptable.

Although, the validated method thus provides a solid approach allowing very little variation in data acquisition (i.e., temperature and LF) and data analysis (i.e., setting of the meniscus), it was nevertheless considered relevant to include a second analyst in the validation study as to include potential variation from sample preparation into the uncertainty budget of the method. Between-sample variation, which can be operator-dependent, may be introduced particularly during sub-sampling and diluting of the as-received laboratory sample, as well as filling of the sample cuvettes. Only for NS-0100A, one-way ANOVA flagged a statistically significant difference (*p* ≤ 0.05) between the group means. Since all other method parameters were fixed, it is assumed that the difference was caused mainly due to sample preparation. Compared to the silica CRMs (ERM-FD101b and ERM-FD102), which were analyzed as-received, the highly concentrated NS-0100A had to be diluted in purified water. The ‘NS’-type RMs come in small bottles with ophthalmic dropper tip fitments and the dosing of the generated droplets is less accurate than for weighing or pipetting. Nevertheless, as the deviating group mean was only 1% above the grand average of the dataset of 20 replicate results, the data was retained. Based on these results, it is concluded that the validated CLS method is also robust against different analysts, provided they are appropriately trained.

### 4.7. Selectivity

The validated method cannot discriminate on particle composition. However, it is selective in terms of its separation efficiency with respect to particle size (and particle density). The validated method could accurately measure the local maxima of the different particle populations of all, except one, prepared mixtures (Table 7 and Figure 1). For these samples, the peaks were baseline resolved (*R*_s_ > 1.5) and the ratio between the diameters of the large and small particles ranged between 1.3 and 2.0. For the mixture prepared from NS-0100A and ERM-FD101b, with a ratio close to 1, the light extinction-weighted PSDs were monomodal instead of bimodal. Although, these PSDs were very narrow and their modes agreed statistically with the certified value of ERM-FD101b, the modal values (i.e., 90 nm) determined for the mixture are significantly larger than those determined for the PSDs of ERM-FD101b alone (i.e., 83 nm). The single peak of the PSDs of the mixture is in fact a convolution of the ERM-FD101b and NS-0100A populations.

From the results of the monodisperse materials, it was concluded that silica particles with nominal diameters smaller than 50 nm could not be accurately measured. Therefore, the results obtained for the bimodal CRM, ERM-FD102, could not be used in the evaluation of the peak resolution.

### 4.8. Measurement Uncertainty

The relative expanded measurement uncertainties (*U*) were estimated by combining the individual relative standard uncertainties from repeatability (*u*_r_), intermediate precision (*u*_ip_) and trueness (*u*_t_), and by applying a coverage factor, *k* = 2 (Equation (7)). As evident from Figure 2, the relative average expanded measurement uncertainty, which does neither change with particle size nor with measurand, is dominated by the uncertainty contribution from trueness. Indeed, since the size of the silica nanoparticles with nominal diameter <50 nm of CRMs ERM-FD100 and ERM-FD304 were beyond the method’s working range, it was decided to use the uncertainty of the certified value of ERM-FD101b as generic contribution in all trueness assessments. The relative standard uncertainties estimated for repeatability and intermediate precision are negligible compared to the trueness uncertainty. The extent of the latter is mainly due to relative standard uncertainty (4.6%) of the certified Stokes diameter. This uncertainty may be considered large, but one should be aware that it consists of numerous contributions which were carefully estimated during the production of ERM-FD101b [43]. In addition to the traditional contributions related to the homogeneity, stability, and characterization of the CRM, *u*_CRM_ also covers uncertainty contributions from the effective density of silica nanoparticles and from polyvinyl chloride (PVC) reference particles used by the majority of the laboratories for calibration of the disc-CLS methods during the characterization study. For the latter, the uncertainties related to the assigned values for particle size (~20%) and effective density (~26%) are significant contributors to *u*_CRM_. Thus, indirectly, they do provide a conservative dimension to the uncertainties estimated in the present validation study. Despite this overestimation, the relative expanded uncertainties of about 10% (Figure 2) are of the same order of magnitude as the measurement uncertainties of size results of silica (nano)particles measured with other techniques, such as particle tracking analysis (11%) [19], disc-CLS (16%) [13], dynamic light scattering (6%) [13] and transmission electron microscopy (7%) [12].

## 5. Conclusions

In this work, a user-friendly cuvette-CLS method for the determination of modal, median and harmonic mean values of light extinction-weighted PSDs of silica (nano)particles in aqueous dispersions was developed and validated in-house. The methodology requires minimal sample preparation (as it can measure as-received suspensions) and employs a robust measurement procedure that successfully passed the validation process. The development and validation experiments were conducted on a selection of CRMs and RMs and binary mixtures of the materials consisting of near-spherical silica particles with diameters in the range of 20 nm to 200 nm and particle mass fractions in the range of 0.1 g/kg to 10 g/kg, according to a nested experimental design.

The results obtained on the monomodal CRMs and RMs demonstrated that the method is suitable to determine the size of silica particles with nominal diameters in the range of 50 nm to 200 nm within a relative expanded uncertainty of 10% at a confidence level of approximately 95% and with particle mass fractions in the range of 0.1 g/kg to 2.5 g/kg. For this size range, the method yielded an average repeatability and intermediate precision of 0.6% and 0.2%, respectively. Finer fractions until 20 nm could be detected but not quantified reliably. Due to the absence of suitable silica CRMs with particle diameters >100 nm, the trueness of the method could only be assessed using a single CRM ERM-FD101b. The relative standard uncertainty for trueness was estimated at 4.6%. Bimodal sample mixtures with size ratios ≥1.3 were satisfactorily discriminated as well.

The comparison of the validated cuvette-CLS method with the more popular disc-CLS method shows its advantages such as operational simplicity, elimination of calibration with reference particles, and high throughput as up to 12 samples can be analyzed simultaneously. The latter may compensate for the long measurement time (i.e., 15 h), compared to other techniques such as DLS or disc-CLS.

The accuracy of particle size results from CLS techniques depends to a large extent on the accuracy of the effective density of the particles. Both the cuvette- and disc-CLS methods have the potential to simultaneously determine the size and effective density of particles by employing spin fluids of different density [16,44,45]. The integration of a validated density method in the presented particle size analysis method will further improve measurement accuracy by lowering the measurement uncertainty.

The results and measurement uncertainties described and discussed in the present contribution may be used by other laboratories to help them in estimating the uncertainties of their cuvette-CLS results.

## Figures and Tables

**Table 1:** Colloidal silica (certified) reference materials used during method development and validation, and their relevant properties.

Code	Reference Particle Diameter (nm)	Mass Fraction in Test Sample (g/kg)	Density (g/cm^3^)	Metrological Status
NS-0020A	19.8 ± 0.5	3.6	1.9	RM
ERM-FD100	20.1 ± 1.3	10	2.3	CRM
ERM-FD304	33.0 ± 3.0	2.5	2.3	CRM
NS-0050A	49.7 ± 1.2	1.8	1.9	RM
ERM-FD101b	87 ± 8	2.5	2.0	CRM
NS-0100A	99.1 ± 2.4	1.3	1.9	RM
ERM-FD305 ^1^	135	1.5	2.0	RM
ERM-FD306 ^1^	135	0.1, 0.15, 1.5, 10	2.0	RM
NS-0150A	146 ± 4	1.3	1.9	RM
NS-0200A	206 ± 5	1.1	1.9	RM
ERM-FD102	23.9 ± 2.0 and 88 ± 7	8.8	2.0	CRM

^1^ Certified reference material for zeta potential measurements, particle size value is an indication only.

**Table 2:** Specifications of in-house prepared bimodal colloidal silica samples

Nominal Particle Diameters (nm)	Monodisperse Silica (C)RMs		Total Mass Fraction in Test Sample (g/kg)	Nominal Mass Fraction (g/kg)	
	Fraction 1	Fraction 2		Fraction 1	Fraction 2
135 + 200	ERM-FD305	NS-0200A	1.3	1.5	1.1
100 + 200	NS-0100A	NS-0200A	1.2	1.3	1.1
100 + 135	NS-0100A	ERM-FD305	1.4	1.3	1.5
80 + 135	ERM-FD101b	ERM-FD305	2.0	2.5	1.5
80 + 100	ERM-FD101b	NS-0100A	1.9	2.5	1.3
50 + 100	NS-0050A	NS-0100A	2.6	3.9	1.3
50 + 80	NS-0050A	ERM-FD101b	3.2	3.9	2.5

**Table 3:** Cuvette-CLS settings and conditions applied for method validation

Method Parameters	Parameter Levels
Type of sample	Colloidal silica
Type of dispersant	Aqueous solution
Dispersant viscosity	0.8927 mPa s
Temperature	25 °C ± 1 °C
Particle size ^1^	50 nm to 200 nm
Particle shape	Equiaxial
Effective particle density	1.9 g/cm^3^ to 2.3 g/cm^3^
Particle mass fraction	0.1 g/kg to 2.5 g/kg
Type of PSD	Mono- and bimodal
Type of signal weighting	Light extinction
Light factor (LF)	0.25 to 1.0
Angular speed	4000 rev/min
Type of cuvette	2 mm and 10 mm polycarbonate
Light source	LED 470 nm
Data acquisition programme	1 cycle at 15 s interval 47 cycles at 5 s interval 110 cycles at 25 s interval 340 cycles at 150 s interval
Analysis mode	Constant position
Background subtraction	Transmission profile of supernatant
Measurement positions	(123.0 ± 0.5) mm, (125.0 ± 0.5) mm and (127.0 ± 0.5) mm

^1^ Harmonic mean, mode and median diameter of a lognormal function fitted to the light extinction-weighted particle size distribution.

**Table 4:** Results for repeatability and intermediate precision

Material	Mean Measured Diameter (nm)			*RSD*_r_ (%)	*RSD*_ip_ (%)	*u*_prec_ (%)
	Median	Harmonic Mean	Mode			
NS-0020A	17.0	17.0	17.0	2.0	ND	NA
ERM-FD100	16.3	16.3	16.3	2.8	0.1 ^1^	1.4
ERM-FD304	28.3	27.8	27.4	4.9	0.9	2.6
NS-0050A	45.6	45.6	45.6	0.8	0.3	0.5
ERM-FD101b	82.9	82.9	82.9	0.2	<0.1 ^1^	0.1
NS-0100A	95.1	95.1	95.1	0.4	0.4	0.5
ERM-FD305	142.1	140.0	137.8	0.5	0.3	0.4
NS-0150A	136.8	136.7	136.8	0.5	0.2	0.3
NS-0200A	191.7	191.3	191.0	1.0	0.5	0.7

^1^ Calculated as *RSD*_ip_^*^; ND, not determined; NA, not applicable.

**Table 5:** Results of the trueness assessment

(C)RM	Δ_m_ (nm)	*u*_meas_ (nm)	*u*_CRM_ (nm)	*u*_t_ (nm)	*RD* (%)	Significant Bias ^1^?
ERM-FD100	3.8	0.2	0.7	0.7	19	Yes
ERM-FD304	5.6	0.7	1.5	1.7	17	Yes
NS-0050A	4.1	NA	NA	NA	8	No
ERM-FD101b	4.1	0.1	4.0	4.0	5	No
NS-0100A	4.0	NA	NA	NA	4	No
NS-0150A	9.2	NA	NA	NA	6	No
NS-0200A	15.0	NA	NA	NA	7	No

^1^ Confidence level of approximately 95%. NA, not applicable.

**Table 6:** Results of robustness testing

Parameter	Level	Mode (nm)	Harmonic Mean (nm)	Median (nm)
***ERM-FD305***				
Temperature (°C)	24	139.9	142.7	145.5
	25 (default)	139.6	142.3	144.9
	26	138.6	141.3 ^1^	143.8 ^1^
Light factor	0.25	139.1	141.2	143.9
	0.7 (default)	139.6	142.3	144.9
	1.0	139.4	141.7	144.2
Meniscus	First profile	138.6	140.8	143.1
	Middle profile	138.7	140.9	143.2
	Last profile (default)	138.6	140.8	143.1
***ERM-FD101b***				
Operator	Operator 1	82.9	82.9	82.9
	Operator 2	83.0	83.0	83.0
***ERM-FD102***				
Operator	Operator 1	89.2	89.0	89.8
	Operator 2	86.9	89.7	90.4
***NS-0100A***				
Operator	Operator 1	94.8 ^1^	94.8 ^1^	94.8 ^1^
	Operator 2	95.5 ^1^	95.5 ^1^	95.5 ^1^

^1^ Significant at 95% confidence level.

**Table 7:** Stokes diameters of local maxima (mean ± standard deviation) and resolution values of bimodal peaks of light extinction-weighted PSDs of bimodal colloidal silica samples

Sample	Peak 1 (nm)	Peak 2 (nm)	Ratio	*R*_s_
ERM-FD305 + NS-0200A	133 ± 4	196 ± 1	1.5	4.8
NS-0100A + NS-0200A	101 ± 2	195 ± 2	1.9	7.5
NS-0100A + ERM-FD305	103 ± 2	131 ± 5	1.3	4.1
ERM-FD101b + ERM-FD305	88.6 ± 0.4	132 ± 2	1.5	4.6
ERM-FD101b + NS0100A	<LOQ	<LOQ	NA	NA
NS-0050A + NS-0100A	46.4 ± 0.2	94.4 ± 0.2	2.0	5.9
NS-0050A + ERM-FD101b	51 ± 1	83.4 ± 0.5	1.6	9.5
ERM-FD102	88 ± 4	< LOQ	NA	NA

NA, not applicable.

## Supplementary Materials

The following are available online at https://www.mdpi.com/1996-1944/13/17/3806/s1, Figure S1: Light transmission profiles obtained for ERM-FD305 and using polycarbonate cuvettes of two different optical path lengths: (a) 2 mm, (b) 10 mm; Figure S2: Transmission signals obtained for ERM-FD305, without and after dilution with purified water: (a) 1.5 g/kg (without dilution), (b) 1.0 g/kg, (c) 0.5 g/kg; Table S1: Effect of cuvette type on measured particle size of ERM-FD305; Table S2: Effect of dilution on measured particle size; Table S3: Sedimentation times obtained for silica RMs of different particle sizes; Table S4: Data acquisition program for silica particles (*x*_St_ = 20 nm to 200 nm); Table S5: Effect of light factor (LF) on measured particle size of selected CRMs; Table S6: Effect of setting the meniscus based on the first and last transmission profiles; Table S7: Effect of measurement position on calculated particle size results for ERM-FD305; Table S8: Effect of background subtraction procedure (blank sample vs. last profile) on measured particle size of selected CRMs.

## Abbreviations

ANOVA	analysis of variance
AUC	analytical ultracentrifugation
CCD	charge-coupled device
CLS	centrifugal liquid sedimentation
CRM	certified reference material
ISO	International Organization for Standardization
*k*	coverage factor
LF	light factor
LLOD_d_	lower limit of detection, in terms of Stokes diameter
LLOQ_d_	lower limit of quantification, in terms of Stokes diameter
*MSB*	mean squares between groups
*MSW*	mean squares within groups
*n*_r_	number of replicates within groups
PVC	polyvinyl chloride
*RD*	relative difference
RM	reference material
*R*_s_	resolution factor
*RSD*_r_	relative standard deviation for repeatability
*RSD*_ip_	relative standard deviation for intermediate precision
*RSD*_ip_^*^	relative standard deviation for intermediate precision (if *MSB* < *MSW*)
TC	Technical Committee
ULOQ	upper limit of quantification
*u*_CRM_	uncertainty of certified value
*u*_prec_	precision uncertainty
*u*_t_	trueness uncertainty
*W*_1/2,1_	width of peak 1 at half-height
*W*_1/2,2_	width of peak 2 at half-height
*X*_c1_	local maximum of peak 1
*X*_c2_	local maximum of peak 2
*y*_m_	arithmetic average calculated from replicate results
Δ_bias_	experimental bias

## Appendix A

**Table A1:** Performance parameters and criteria considered during validation study.

Performance Parameter	Experiment	Performance Criterion
Robustness	Small deliberate changes applied during data acquisition and data analysis (method development) and measurements conducted by two analysts (method validation)	Results not significantly ^1^ different
Linearity and calibration	NA	NA
LLOD_d_/LLOQ_d_/ULOQ_d_	Nested experiments on different types of colloidal silica, mainly varying with respect to particle size (range of 20 nm to 200 nm in diameter)	LLOD_d_: Significant ^1^ difference and/or relative standard deviation for repeatability (*RSD*_r_) ≤ 5.0% LLOQ_d_: Not significantly ^1^ different and *RSD*_r_ ≤ 1.5% ULOQ_d_: *RSD*_r_ ≤ 1.5%
Working range	Particle size: Nested experiments on different types of colloidal silica RMs, mainly varying with respect to particle size (range of 20 nm to 200 nm in diameter) Particle mass fraction: Dilution series of different silica RMs	Particle size: No significant ^1^ bias and accurate within ≤ 1.5% Particle mass fraction: One-way ANOVA or *t*-test, no significant ^1^ differences
Selectivity	Mixing of different monodisperse colloidal silica RMs	Reliable measurement of particle populations which differ in size by a factor 4
Repeatability and intermediate precision	Nested experiments (i.e., five days with four replicates per day) on different types of colloidal silica RMs, mainly varying with respect to particle size (range of 20 nm to 200 nm in diameter)	One-way ANOVA, *RSD*_r_ ≤ 1.5%, no significant ^1^ differences among groups (days)
Trueness	Nested experiments (i.e., five days with four replicates per day) on different types of colloidal silica CRMs, mainly varying with respect to particle size (range of 20 nm to 80 nm in diameter)	No significant ^1^ bias
Measurement uncertainty	Combination of top-down and bottom-up approach	*U*^1^ ≤ 15%

NA, not applicable; *U*, expanded uncertainty; ^1^ confidence level of 95%.
