# Supplementary material for: Validation of a Homogeneous Incremental Centrifugal Liquid Sedimentation Method for Size Analysis of Silica (Nano)particles
Source: Materials (Basel). 2020 Aug 28;13(17):3806. doi: 10.3390/ma13173806 (PMC7503812; doi:10.3390/ma13173806)
Supplement: Supplementary file 1 [file materials-13-03806-s001.pdf]

materials

Supplementary Materials

Validation of a Homogeneous Incremental Centrifugal Liquid Sedimentation Method for Size Analysis of Silica (Nano)particles

Jesús Manuel Antúnez Domínguez, Yannic Ramaye, Marta Dabrio and Vikram Kestens,*

European Commission, Joint Research Centre (JRC), 2440 Geel, Belgium; jesusantunezdominguez@hotmail.com (J.M.A.D.); yannic.ramaye@ec.europa.eu (Y.R.); marta.dabrio@ec.europa.eu (M.D.)

* Correspondence: vikram.kestens@ec.europa.eu; Tel.: +32-14-571614 (V.K.)

1. Results and Discussion Method Development

1.1. Type of Sample Cuvette

Figure S1 presents the light transmission profiles acquired for ERM-FD305 analyzed with two different rectangular polycarbonate cuvettes with external dimensions of (2 × 8) mm and (10 × 8) mm. The abscissa corresponds to the optical detection zone expressed as the lateral distance from the center of rotation. The set of profiles is read from the left to the right, as this is the sedimentation direction of the particles (cuvettes are placed horizontally into the rotor). The starting point of the measurement is given by a sharp vertical jump that represents the sample's meniscus, or the boundary between the gas phase (left of the meniscus) and the liquid phase (right of the meniscus). Throughout a sedimentation process, a second optically distinguishable liquid/liquid boundary is established during centrifugation between the particle-free liquid (supernatant) and the suspension, whereby the profiles gradually move from the bottom to the top. Ultimately, the particles sediment out in the sedimentation zone (about 2 mm thickness for the given example) near the cuvette bottom (x-value of 130 mm).

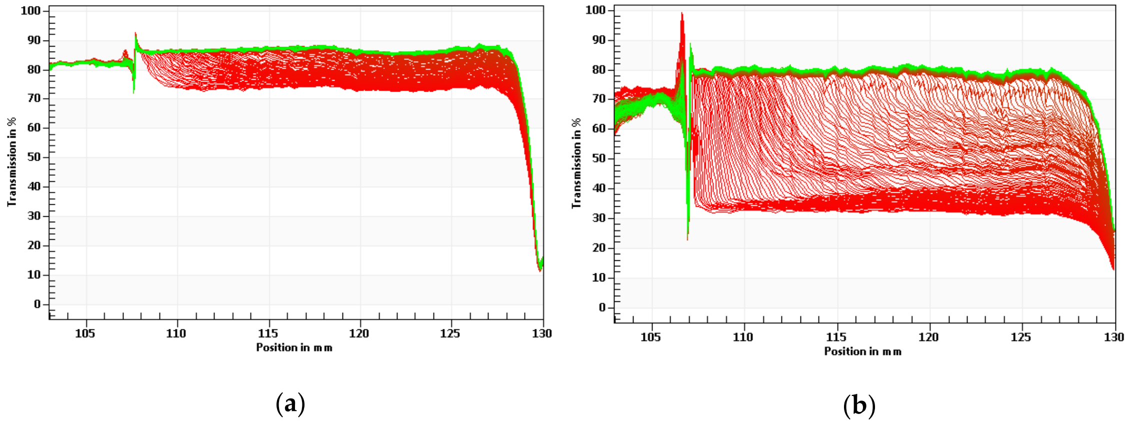

Figure S1. Light transmission profiles obtained for ERM-FD305 and using polycarbonate cuvettes of two different optical path lengths: (**a**) 2 mm, (**b**) 10 mm.

The two fingerprints (or sets of transmission profiles) obtained for ERM-FD305, and depicted in Figure S1, significantly differ depending on the type of cuvette used. In case of the slim cuvette (a), all profiles are grouped within the transmission range of 70% to 90%. For the larger cuvette with an optical path length of 10 mm, profiles were collected between 30% and 80% of transmission. The instrument manufacturer recommends that the transmission signal of the first profiles acquired first should be in the range of about 30% to 60%, as this will allow that the sedimentation information

embodied in the profiles will be sufficiently spread and accessible for analysis. For dilute and highly transparent samples, the initial transmission level often exceeds 60%, thereby squeezing all profiles in a relatively narrow range. In that case, the use of cuvettes with a longer optical path length will reduce the initial transmission while turbid samples will profit from cuvettes with shorter path lengths.

ERM-FD305 has a transparent appearance and the fingerprint obtained with the larger cuvette type therefore contains more information than in case of the slim cuvette. Nevertheless, as shown from the results listed in Table S1, the two cuvettes gave similar particle size results. Using the Student's *t*-test the results for the median diameter were found statistically different at a confidence level of 95%. However, this difference is considered technically negligible and because no significant difference was found for the two other measurands, it was concluded that both types of cuvettes could be used during the validation study. The ability to use two types of cuvettes increases both the versatility and robustness of the finally validated method.

Table S1. Effect of cuvette type on measured particle size of ERM-FD305.

Optical Path Length (mm)	Median (nm)	Harmonic Mean (nm)	Mode (nm)
2	149	144	141
10	145	142	139

The results shown in Table S1 were obtained using the following method parameters:

- Temperature: 25 °C
- LED source: 470 nm
- Speed: 4,000 rev/min
- Light factor: 1.0
- Cycle configuration:
    - 60 profiles at 5 s interval
    - 200 profiles at 10 s interval
    - 100 profiles at 30 s interval
- Constant mode analysis
- Nodes at 123 mm, 125 mm and 127 mm
- Node width: 1.0 mm

1.2. Particle Mass Fraction and Dilution

In the previous section, it was shown that the transmission signal of the (first) transmission profiles can be reduced (or increased) by using a cuvette with a different optical path length. If a sample is too turbid, meaning that the level of the first profile is significantly below 30% of transmission, dilution may be applied. Sometimes, dilution may also be required if very little amount of sample is available.

To investigate the possible effect of dilution, different dilution series were prepared from ERM-FD100, ERM-FD304, ERM-FD101b, ERM-FD306 and ERM-FD305. For each material and particle mass fraction, measurements were performed in quadruplicate, using purified water or borate buffer at pH 8–9 (for ERM-FD306) as diluting agent, and applying the measurement conditions given in Section 1.1. All samples were contained in cuvettes with an optical path length of 10 mm. As can be seen from the obtained results, shown in Table S2, diluting the selected CRMs with purified water did not result in significantly different particle size results. However, for ERM-FD306 a systematic trend of decreasing calculated median and mean Stokes diameters and increasing particle mass fractions (> 1.0 g/kg) can be seen. These results also support the reliability of the particle size results obtained on the non-certified RMs during the validation study, for which a forced dilution with purified water was required.

The effect of dilution on the transmission level of the profiles, obtained for ERM-FD305, is shown in Figure S2.

Table S2. Effect of dilution on measured particle size.

Sample	Mass Fraction (g/kg)	Median (nm)	Harmonic Mean (nm)	Mode (nm)
ERM-FD100	10	17	17	17
	6.8	17	17	17
	3.4	19	19	19
ERM-FD304	5.0	28	28	28
	3.4	29	29	29
	1.6	30	27	25
ERM-FD101b	2.5	83	83	83
	1.7	82	82	82
	0.8	82	82	82
ERM-FD305	1.5	141	139	137
	1.0	140	138	135
	0.5	137	138	137
ERM-FD306	10.0	142	139	137
	5.0	145	143	140
	1.0	147	143	140
	0.15	148	143	138
	0.1	147	144	139

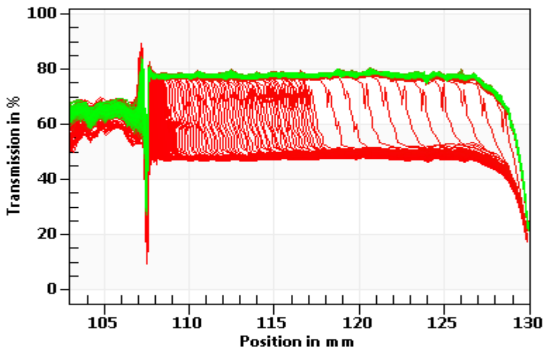

(a)

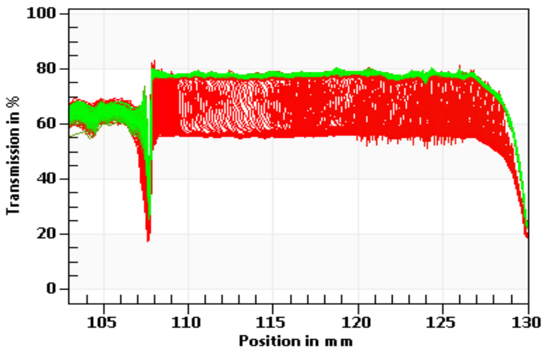

(b)

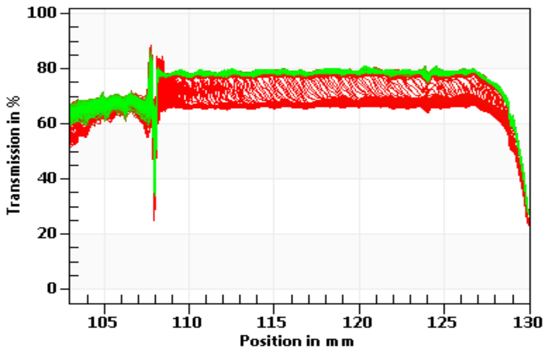

(c)

Figure S2. Transmission signals obtained for ERM-FD305, without and after dilution with purified water: (**a**) 1.5 g/kg (without dilution), (**b**) 1.0 g/kg, (**c**) 0.5 g/kg.

1.3. Data Acquisition Time

One of the most crucial parameters in analytical centrifugation is the measurement or data acquisition time. The data acquisition time must be optimized based on the (expected) size and effective density of the particles to be analyzed. Acquisition times set too short will result in particle size distributions (PSDs) that are incomplete and biased. On the other hand, acquisition times set unnecessarily long reduce the sample throughput.

The LUMiSizer 650 software allows programming data acquisition cycles at different time intervals between each profile. An example of such program specifically valid for ERM-FD305 is given in Section 1.1 of this Supplementary Materials. In this case, the short time interval of 5 s will allow detection of potentially present large particles, including agglomerates, while the 10 s and 30 s intervals will collect sedimentation data from the main particle population as well as very tiny particles at the very extreme of the size distribution.

Ideally, the centrifugation program is optimized for a material of specific particle size and particle density. Instead, in our study we aimed at developing a more versatile method that allows analyzing silica particles of 20 nm up to 200 nm in diameter. As a result, the overall measurement duration must be, on the one hand, sufficiently long as to detect the smallest nanoparticles, but, on the other hand, the time intervals must allow capturing also the largest particles of 200 nm. During the method development stage, individual programs (in terms of acquisitions cycles and time intervals) were first established for selected RMs covering the intended size range of 20 nm to 200 nm. Table S3 shows the experimentally determined times needed for a complete sedimentation at a maximum centrifugation speed of 4,000 rev/min.

Table S3. Sedimentation times obtained for silica RMs of different particle sizes.

Reference Material	Sedimentation Time (min)
ERM-FD100	720
ERM-FD304	300
ERM-FD101b	50
ERM-FD305	30
NS-0200A	15

Based on the individual sedimentation programs, a generic program consisting of different data acquisition or measurement cycles was established (Table S4).

Table S4. Data acquisition program for silica particles (x_{St} = 20 nm to 200 nm).

Number of Measurements	Time Interval (s)	Acquisition Time (min)	Indicative Particle Size (x_{St}) Range (nm)
1	15	0.25	Until rotor reaches maximum speed
47	5	4	> 200 nm
110	25	46	80–200
340	150	850	20–80

1.4. Light Factor

The light factor (LF) is a dimensionless instrumental parameter value that is related to the intensity of the incident light beam. According to the instrument manufacturer, a default value of 0.7 is suitable for most types of colloidal samples. For dilute samples with a highly transparent appearance, only a limited fraction of the incident light will be extinct by the sample suspension and the high intensity of the light passing through the sample may cause saturation of the photodetector.

This optical phenomenon can be avoided by applying a lower LF value. On the other hand, higher LF values (e.g., up to 2.00) may be used for turbid samples, thereby improving the signal-to-noise ratio of the measurement signal.

The colloidal silica samples subject to the present study were very dilute and had a *quasi* (semi-)transparent appearance. Therefore, only the possible effect of a reduced LF value (i.e., 0.25) was investigated. Samples of ERM-FD100, ERM-FD304, ERM-FD101b and ERM-FD305 were analyzed in quadruplicate. Measurements were performed using a common program at 4,000 rev/min. During the first test series, the LF was set to its recommended default value of 0.7. In the second test series, the centrifuged and analyzed samples were re-dispersed using a vortex homogenizer and re-measured using the same program but applying a LF value of 0.25, instead. The cuvette-CLS method is non-destructive and, if samples can be effectively re-dispersed, which was the case for the abovementioned CRM, then, re-measuring samples avoids the introduction of potential sample-to-sample variability into the measurement result. The averages of the four replicate results, expressed for the main central tendencies of the light extinction-weighted PSDs, are given in Table S5. Note that at this stage of the method development study, the results of the two measurement sets were compared only relatively to one other and they were not compared with the CRMs' certified values.

As can be concluded from the obtained results, and following significance testing at a confidence level of 95%, the reduced LF value did not have a significant impact on the main central tendency values of the light extinction-weighted PSDs obtained for the tested materials.

Table S5. Effect of light factor (LF) on measured particle size of selected CRMs.

Certified Reference Material	LF	Median (nm)	Harmonic Mean (nm)	Mode (nm)
ERM-FD100	0.25	16.7	16.7	16.7
	0.7	17.0	17.0	17.0
ERM-FD304	0.25	28.8	29.8	28.7
	0.7	27.7	26.6	27.5
ERM-FD101b	0.25	81.4	81.4	81.4
	0.7	81.1	81.1	81.1
ERM-FD305	0.25	143.9	141.2	139.1
	0.7	144.9	142.3	139.6
	1.0	144.2	141.7	139.4

1.5. Measurement Position and Meniscus

When the centrifugation program has completed, the particle sedimentation data stored within the acquired light transmission-based fingerprints can be converted into meaningful light extinction-weighted PSDs using Stokes' law. The data conversion requires selecting a defined region of the fingerprint that is at a sufficiently distance from the sample's meniscus, and which contains the sedimentation data of the particles intended to be analyzed. To increase the robustness of the measurement result, independent measurements are conducted at three different positions along the fingerprint and the three obtained PSDs are averaged creating one final PSD. This data evaluation approach includes defining the meniscus, subtracting data from either a blank sample or from the supernatant, and defining the actual measurement positions.

The meniscus of the sample, or symbol S in Equation (1) provided and discussed in the article, which corresponds to the sample filling height, is a critical input parameter whose value directly affects the result of the measurement. As can be seen from the fingerprint examples shown above, the meniscus of the sample during centrifugation can be subject to some degree of variation, as the local minima of the individual profiles do not always exactly overlap. Hence, defining the position of the meniscus is operator-dependent and its associated uncertainty should be determined and included in the overall uncertainty budget of the CLS method. The actual zone over which the meniscus can vary depends on the quality of the sample being analyzed. At least for monodisperse samples such as ERM-FD305, the effect of the meniscus setting showed to be negligible when using

either the first, the middle or the last acquired transmission profile (Table S6). Nevertheless, to eliminate the precision component from the meniscus setting process it was decided that the meniscus will be set using the last acquired transmission profile. The remaining trueness component is then assumed be covered by the overall trueness of the validated method.

Table S6. Effect of setting the meniscus based on the first and last transmission profiles.

Transmission Profile	Median (nm)	Harmonic Mean (nm)	Mode (nm)
1	143.1	140.8	138.6
249 (middle)	143.2	140.9	138.7
498 (last)	143.1	140.8	138.6

Using the previous experiments on ERM-FD305, the effect of the measurement position was investigated. Light extinction-weighted PSDs were calculated using different measurement positions that were selected at distances in the range of 110 mm to 127 mm from the center of revolution. As can be seen from the obtained results (Table S7), the calculated particle diameter values systematically increase with the distance from the center of rotation. The difference in particle size calculated for the two extreme positions is about 3%. As recommended by the instrument manufacturer, by averaging over three separate measurement zones located at a distance of (123.0 ± 0.5) mm, (125.0 ± 0.5) mm and (127.0 ± 0.5) mm from the center of revolution, the effect of the positive trend is flattened.

Table S7. Effect of measurement position on calculated particle size results for ERM-FD305.

Measurement Position (mm)	Median (nm)	Harmonic Mean (nm)	Mode (nm)
110 ± 0.5	141.1	138.7	136.4
115 ± 0.5	141.5	139.7	138.0
120 ± 0.5	142.3	140.3	138.2
124 ± 0.5	143.0	140.9	138.8
125 ± 0.5	143.1	141.0	139.2
126 ± 0.5	144.2	141.9	139.4
127 ± 0.5	145.0	142.6	140.9

Finally, the process of transforming the light transmission profiles into a light extinction-weighted PSD requires subtraction of the time-independent background signal, caused by possible inhomogeneity in the optical components. This can be conveniently accomplished by running samples of the original dispersant medium simultaneously with the test samples. If the original dispersing medium or its chemical recipe is not available, which is often the case, then the optical signal of the last acquired transmission profile, which is assumed that it corresponds to the supernatant or the particle-free liquid, can be used alternatively. Quadruplicate experiments on CRMs were conducted to compare results obtained by both measurement approaches.

From the average results calculated from the quadruplicate results (Table S8), it can be concluded that for ERM-FD304 and ERM-FD101b, either approach gave equal results. For ERM-FD305, the particle size results calculated following subtraction of a blank sample resulted in systematically higher values. Given an expanded uncertainty of about 10% (see validation results), the systematic difference can, however, be regarded as technically irrelevant. It must be noted that this stage only envisaged evaluating the relative differences between results obtained with the two background subtraction methodologies. Evaluation of the method's accuracy, i.e. comparing the experimental results with the certified values, was not aimed yet.

Table S8. Effect of background subtraction procedure (blank sample vs. last profile) on measured particle size of selected CRMs.

Certified reference Material	Median (nm)	Harmonic Mean (nm)	Mode (nm)

	**Last Profile**	**Blank**	**Last Profile**	**Blank**	**Last Profile**	**Blank**
ERM-FD304	28.3	28.3	28.3	28.3	28.3	28.3
ERM-FD101b	82.3	82.3	82.3	82.3	82.3	82.3
ERM-FD305	141.1	142.2	138.4	139.4	134.8	136.4
